# Association between external eating and dietary patterns among middle school students in Chongqing, China

**DOI:** 10.3389/fnut.2025.1593587

**Published:** 2025-07-23

**Authors:** Yingjiao Deng, Lingxi Zhao, Linlu Guo, Yu Zhang, Yuanyuan Lan, Youtao Mou, Wenhua Ran, Ling Liu, Zumin Shi, Yong Zhao

**Affiliations:** ^1^School of Public Health, Chongqing Medical University, Chongqing, China; ^2^Research Center for Medicine and Social Development, Chongqing Medical University, Chongqing, China; ^3^Research Center for Public Health Security, Chongqing Medical University, Chongqing, China; ^4^Sichuan and Chongqing Regional Nutrition Innovation Platform, School of Public Health, Chongqing Medical University, Chongqing, China; ^5^Department of Clinical Nutrition, Chengdu Seventh People’s Hospital (Affiliated Cancer Hospital of Chengdu Medical College), Chengdu, China; ^6^Yuzhong District Primary and Middle School Health Care Center, Chongqing, China; ^7^Human Nutrition Department, College of Health Science, Qatar University, Doha, Qatar; ^8^Chongqing Key Laboratory of Child Nutrition and Health, Children’s Hospital of Chongqing Medical University, Chongqing, China

**Keywords:** external eating, dietary patterns, middle school student, exploratory factor analysis, China

## Abstract

**Background:**

The dietary pattern, serving as a comprehensive indicator of the nutritional intake of middle school students, plays a crucial role in guiding their health. External eating, the behavior of eating based on external cues associated with food (e.g., the sight and smell of food), is a key factor influencing the choice of dietary patterns.

**Objective:**

To investigate the dietary patterns of middle school students in Chongqing and analyze the association between external eating and dietary patterns.

**Methods:**

A cross-sectional study was conducted among 6,590 middle school students from 17 districts and counties in Chongqing, selected by stratified cluster sampling. The questionnaire included basic information about the participants, external eating score [based on the Dutch Eating Behaviour Questionnaire (DEBQ)] and dietary frequency, and data were collected via online self-report. Exploratory factor analysis was employed to extract dietary patterns, and multiple linear regression analysis was used to analyze the association between external eating and dietary patterns among middle school students.

**Results:**

The total score for external eating among middle school students in Chongqing ranges from 19 to 27. Three dietary patterns were identified among middle school students: modern dietary pattern, fast-food dietary pattern, and balanced dietary pattern. The results of multiple linear regression analysis showed that, external eating is positively associated with fast-food and balanced dietary pattern after adjusting for confounders [β = 0.006, 95% confidence interval (CI) : (0.002, 0.011), *P* = 0.002; β = 0.005, 95% CI: (0.001, 0.010), *P* = 0.016], external eating was negatively associated with modern dietary pattern [β = –0.005, 95% CI: (–0.009, –0.001), *P* = 0.018].

**Conclusion:**

External eating exhibits a significant negative association with modern dietary pattern among middle school students. Therefore, middle school students should be guided to correctly deal with external eating, so as to establish a reasonable dietary pattern.

## 1 Introduction

Externality theory posits that external environments or stimuli can induce individuals to eat, reducing sensitivity to internal hunger and satiety cues, a phenomenon known as external eating (1). External eating is characterized by eating in response to external food-related cues, such as the sight and smell of food, regardless of internal hunger signals (2). This phenomenon reflects the influence of changes in eating environments, utensils, and dietary patterns (3) and is implicated in the rising prevalence of obesity and food cravings (4). Individual sensitivity to external food cues is a critical factor influencing eating behavior (5), and while behavioral factors shape food choices, they are also significantly influenced by environmental factors (6). Environmental factors are now recognized as major drivers of obesity (7–9). The modern food environment, characterized by a wide variety of palatable and energy-dense foods, often serves as a cue for overeating (10). Additionally, food advertising plays a significant role, with sugary drinks being the most frequently advertised products, while advertisements for vegetables and fruits are scarce (11, 12). This imbalance can contribute to unhealthy eating habits and related health issues among middle school students (13). Previous research has extensively examined the links between eating behaviors and dietary patterns. The Dutch Eating Behaviour Questionnaire (DEBQ) (14) evaluates three key eating behaviors: emotional eating (eating in response to negative emotions), restrained eating (consciously restricting food intake to control weight), and external eating (eating in response to external food cues rather than internal hunger signals). Emotional eating (15) is typically linked to unhealthy dietary patterns, restrained eating (16) can be associated with both healthy and unhealthy eating behaviors depending on context. External eating exhibits a positive association with food craving, which may increase actual food intake without necessarily leading to binge-eating behavior, particularly among middle school students demonstrating weaker inhibitory control (17). Studies have shown that students’ candy intake is positively associated with external eating (18), and those more sensitive to external food cues are at a higher risk of excessive weight gain and obesity (19).

Dietary patterns, which comprehensively measure the quantity, variety, proportion, and frequency of food consumption, provide a holistic assessment of nutrient interrelationships and their impact on health (20). These patterns are widely used in nutritional epidemiology to evaluate individual dietary nutrient intake (21). Middle school is a critical period for the formation of lifelong eating habits, making dietary patterns particularly important for the physical health of students (22). A study in China identified five distinct dietary patterns among children and adolescents aged 7–17: DP1 (animal products and vegetables), DP2 (condiments), DP3 (fruits and junk food), DP4 (cereals and tubers), and DP5 (beans) (22). Similarly, a study in Luzhou city revealed three dietary patterns among adolescents: vegetable and fruit, fast food, and staple meat patterns (21). The choice of dietary patterns is influenced by various factors, including eating behavior, geographic location, and cultural practices (23, 24). External stimuli such as visual and olfactory cues, as well as food advertising, can significantly impact individual eating behaviors, highlighting the importance of external eating in shaping dietary patterns (25).

External eating has been positively associated with unhealthy food consumption (26). As unhealthy foods like sugary drinks and fried snacks become more prevalent in the lives of middle school students, environmental cues (i.e., external eating) have been linked to higher intake of high-energy snacks and sugar-sweetened beverages (27). Given that the “fruit and junk food” dietary pattern includes high-energy snacks and sugary drinks, we hypothesize that external eating is negatively associated with the adoption of healthier dietary patterns. However, there is limited research on the association between external eating and dietary patterns among middle school students, both domestically and internationally. Chongqing’s unique mountainous terrain and the wharf culture at the confluence of the two rivers have given rise to fast-paced eating habits, which have combined to produce the distinctive Chongqing dietary pattern (28). In terms of social culture, Chongqing is a city of immigrants, who have brought foreign food habits into Chongqing, forming a distinctive Chongqing food culture. Chongqing has both large rural and urban areas, and there are differences in dietary choices between adolescents from urban and rural areas (29). The interaction of many factors makes Chongqing’s dietary patterns irreplaceable and extremely important to study. This cross-sectional study aims to explore this relationship among middle school students in Chongqing, providing a theoretical basis for improving poor eating behaviors and encouraging the adoption of healthier dietary patterns.

## 2 Materials and methods

### 2.1 Study design and sample

Seventeen districts and counties in four areas of the Chongqing region (the main city, the new main city, the northeast of Chongqing, and the southeast of Chongqing) were selected as areas for stratified cluster sampling, and local junior and senior high school students were invited to voluntarily fill out the online questionnaire. The sample size required for the study was calculated using the following sample size formula for cross-sectional surveys:


$$n=\frac{Z_{\alpha }^{2}\times p\,q}{d^{2}}$$

In the above sample size calculation formula, where *n* is the required sample size, the Report on Nutrition and Chronic Disease Status of Chinese Residents (30) states that the obesity rate of children and adolescents in China reaches 19%. Therefore, we set *p* to 0.19, set α = 0.05 (bilaterally), *Z*α = 1.96, and d denotes the allowable error, using *d* = 0.1p, *q* = 1−0.19 = 0.81, and *d* = 0.019, so by substituting the above data into equation *n*, the sample size was calculated to be 984, and the minimum required sample size was calculated to be 1,181, taking into account the 20% non-response rate.

### 2.2 Inclusion and exclusion criteria

The following inclusion criteria were used: (a) students enrolled in secondary schools in Chongqing (junior to senior), (b) students themselves, their parents and class teachers fully understood the purpose of this survey and gave informed consent, and (c) able to complete the survey independently.

The following exclusion criteria were used: (a) students with endocrine diseases affecting growth and development, as well as psychological problems and mental illnesses and (b) those who are unable to cooperate in completing the survey due to illness or other factors.

**FIGURE 1:**
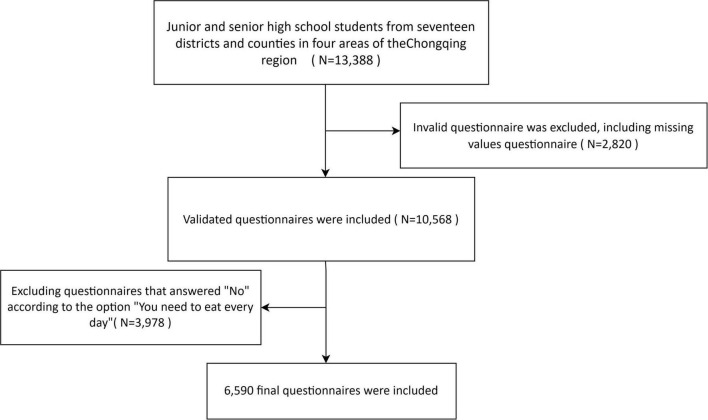
Flow chart for inclusion of study participants.

A total of 13,388 questionnaires were collected in the back office, 2,820 invalid questionnaires were excluded, and 10,568 questionnaires were included, 3,978 excluding questionnaires that answered “No” according to the option “You need to eat every day,” 6,590 final questionnaires were included (see Figure 1).

The study was reviewed and approved by the Ethics Committee of Chongqing Medical University (approval number: 2021041). The link to the online survey was sent to school administrators through the Chongqing Municipal Education Commission, who in turn forwarded it to classroom teachers. These teachers then shared the link with their parent contact groups, clearly explaining the purpose and significance of the survey, the principle of voluntary participation (which was not mandatory), and the need to obtain prior parental permission to allow students to access the questionnaire. Students could only access the survey page on their mobile devices after obtaining parental permission. Students who agreed to participate were required to sign an e-consent form before proceeding, while those who opted out were allowed to close the page immediately without any further obligation.

### 2.3 Data collection

A cross-sectional study was conducted through the “Questionnaire Star” online platform, in which the questionnaire “Survey on the Status of External Eating and Dietary Patterns of Middle School Students” was sent to school work groups in 17 districts and counties in Chongqing as a link to be filled out, and the instructions and link were sent by classroom teachers or other teachers in charge to the parents’ groups, with the informed consent of parents and students. After parents and students agreed, the questionnaire was filled out by the students themselves on weekends or during home time after school, with an average filling time of 8 min.

### 2.4 Questionnaire design

The questionnaire was designed on its own by reviewing the literature and consulting with experts, and was adjusted and modified according to the results of the preliminary survey. The external eating scale was based on the Dutch Eating Behavior Questionnaire (DEBQ) developed by van Strien et al. (31). This questionnaire has been proven to be suitable for adolescents in China (32), and its Chinese translation was based on the study by Li et al. (33), which verified that the Chinese version of the DEBQ has good reliability and validity. Similar entries were merged after many discussions among experts in the field of nutrition and health behavior and panelists. The survey data validation showed that the scale has good reliability and validity (Cronbach’s alpha coefficient of 0.87). The qualitative dietary frequency questionnaire was adopted from the dietary frequency questionnaire designed by the Chinese Center for Disease Control and Prevention (CDC). The questionnaire consists of four parts, including basic demographic characteristics, external eating scale, dietary frequency survey, and daily living habits.

The first part included information on basic demographic characteristics such as age, gender, ethnicity, live in school or not, residence, only children or not, parenting mode, parenting education level, height, weight, and body type self-assessment, daily exercise time, daily screen sedentary time. The external eating scale used in the second part, with 10 entries, was scored on a five-point Likert scale, all with positive scores, with total scores between 10 and 50. Higher scores indicate more severe eating behavior problems among middle school students (33). The third section consisted of the Food Frequency Questionnaire (FFQ), which asked the respondents to review the food items they had all consumed in the past month and to estimate the average number of times the food items were consumed. The FFQ included a variety of food items, essentially covering the entire range of foods that students consume in their daily lives. In this study, 70 food items were categorized into 20 food groups based on food characteristics and nutritional structure for inclusion in the study. The fourth section included the number of exercise sessions per week (less than 1 session/1–2 sessions/3–4 sessions/5–6 sessions/>7 sessions), the duration of each session (less than 0.5 h/0.5–1 h/more than 1–1.5 h/more than 1.5–2 h/more than 2 h), the level of fatigue at the time of the physical activity (very mild or mild/shortness of breath, rapid heart rate, and verbal communication is possible/deepening and rapid breathing, heart rate greatly increased, difficulty in verbal communication), daily sleep duration (less than 7 h/7–8 h/more than 8–9 h/more than 9–10 h/more than 10 h), and daily screen sedentary time (less than 2 h/2–6 h/more than 6–8 h/more than 8 h).

### 2.5 Statistical analysis

Data were analyzed using STATA version 18.0 (STATA Corporation, College Station, TX, United States), SPSS version 25.0. Statistical significance was considered when *P* < 0.05 (two-sided). Dietary patterns were extracted by exploratory factor analysis, and when the absolute value was greater than 0.3, the food item was considered to be the main contributor to this dietary pattern. The number of dietary patterns was determined based on eigenvalues ≥1.0 and the interpretability of the patterns. Differences between groups were compared by Chi-square test and *t*-test. Multiple linear regression was used to analyze the differences in external eating among the three different dietary patterns, frequency and proportions (%) were used to describe categorical variables and mean ± SD was utilized to describe continuous variables. We included variables with positive results on univariate analyses (*P*-value < 0.05 variables in Table 3) as confounders in the multivariate analyses.

## 3 Results

### 3.1 Dietary patterns of middle school students

Dietary pattern naming based on the characteristics of the food groups with higher factor loadings can be classified into three types, which are modern dietary pattern, fast-food dietary pattern, and balanced dietary pattern. Together, these three dietary patterns explained 72.6% of the total variation. The cumulative percentage of variance of the three dietary patterns were 13.3%, 24.6%, and 34.7%, respectively. Modern dietary pattern is characterized by a high intake of animal offal, mushrooms and algae, legumes, aquatic products, poultry, and fresh vegetables, and an appropriate intake of eggs; the fast-food dietary pattern is characterized by a high intake of fried dough foods, processed meat products, and sugar-sweetened beverage, desserts/candies, and tea and coffee, with an appropriate intake of animal offal; the balanced dietary pattern is characterized by a high intake of cereals, fresh vegetables, red meat and purified water, with an appropriate intake of tubers, fruits, dairy products, poultry, and eggs (see Table 1).

**TABLE 1:** The factor loading of three dietary patterns and food groups among middle school students in Chongqing.

Food category*	Modern dietary pattern	Fast food dietary pattern	Balanced dietary pattern
Cereals			0.68
Tubers	0.46		0.46
Fried dough foods		0.70	
Legumes	0.59		
Fresh vegetables	0.37		0.67
Mushrooms and algae	0.65		
Fruits			0.41
Red meat	0.34		0.64
Poultry	0.56		0.46
Processed meat products	0.45	0.63	
Animal offal	0.66	0.45	
Aquatic products	0.58		
Dairy products			0.42
Eggs	0.32		0.44
Nuts			
Desserts/candies		0.54	
Dried fruits and vegetables			
Purified water			0.56
Sugar-sweetened beverages		0.70	
Tea and coffee		0.51	
% of cumulative variance	13.3	24.6	34.7

*Only keep the numerical values with a load factor of >0.3.

### 3.2 Basic information of external eating in middle school students in Chongqing

In the External Eating Scale, there are a total of 10 items. For item 1, “When the food tastes good, you will eat more than usual”: 27.7% sometimes do, 27.0% often do, and 22.2% always do. For item 2, “When the smell and appearance of the food are good, you will eat more than usual”: 30.9% sometimes do, 22.2% often do, and 15.4% always do. For item 4, “When you see or smell some delicious food, you crave it”: 27.0% sometimes do, 20.8% often do, and 16.3% always do. For item 6, “When you pass by snack stalls, restaurants, or bakeries, you will buy or store some delicious food”: 27.3% sometimes do, 8.2% often do, and 5.7% always do (see Table 2).

**TABLE 2:** Basic information of external eating in middle school students in Chongqing.

Variables	Number of cases (composition ratio), *N* (%)				
	Never	Now and then	Sometimes	Often	Always
When the food tastes good, you will eat more than usual	373 (5.7)	1,146 (17.4)	1,828 (27.7)	1,779 (27.0)	1,464 (22.2)
When the food smells and looks good, you will eat more than usual	552 (8.4)	1,521 (23.1)	2,034 (30.9)	1,464 (22.2)	1,019 (15.4)
When you start eating, you cannot seem to stop	2,760 (41.9)	1,800 (27.3)	1,206 (18.3)	452 (6.9)	372 (5.6)
When you see or smell some delicious food you are eager to eat it	663 (10.1)	1,700 (25.8)	1,777 (27.0)	1,372 (20.8)	1,078 (16.3)
When you have delicious food you want to eat it right away	906 (13.8)	1,957 (29.7)	1,705 (25.9)	1,100 (16.6)	922 (14.0)
When you pass by a snack bar, restaurant or bakery you buy or store some delicious food	1,303 (19.8)	2,567 (39.0)	1,801 (27.3)	541 (8.2)	378 (5.7)
You can resist the temptation of delicious food			2,078 (31.5)	2,991 (45.4)	1,521 (23.1)
When you see other people (around you, on video, or in pictures) eating, you tend to eat more than usual	2,809 (42.6)	1,996 (30.3)	1,276 (19.4)	279 (4.2)	230 (3.5)
When you prepare meals you tend to eat things (ingredients)	1,444 (21.9)	2,259 (34.3)	1,943 (29.5)	556 (8.4)	388 (5.9)
When you see something that looks delicious, you often feel so hungry that you have to eat it right away	3,140 (47.7)	1,994 (30.3)	969 (14.7)	243 (3.7)	244 (3.7)

### 3.3 Differential analysis of three dietary patterns of middle school students with different demographic characteristics

Among 6,590 middle school students, the age of the students is presented as the median with the interquartile range (IQR). The median age is 15 years. The IQR is from 14 to 16 years, which means that the middle 50% of the participants’ ages fall within this range. The external eating scores are also presented as the median with the IQR. The median score is 23. The IQR ranges from 19 to 27. Those with a modern dietary pattern accounted for 29.9% (1,968 participants) of the total, those with a fast-food dietary pattern accounted for 40.5% (2,668 participants), and those with a balanced dietary pattern accounted for 29.7% (1,954 participants). Compared with students in other dietary pattern groups, students in the balanced dietary pattern group were younger (*P* < 0.001). Compared with girls, boys had a higher proportion in the balanced dietary pattern group, while in the modern dietary pattern group, girls had a higher proportion (*P* < 0.001). Compared with middle school students living in towns, those living in rural areas had a higher proportion in the balanced dietary pattern (*P* < 0.001). Compared with boarding students in other dietary pattern groups, the proportion of non-boarding students was higher in the fast-food dietary pattern group (*P* < 0.001). A lower proportion of students in the modern dietary pattern group met the sleep criterion compared to students in the other dietary patterns group, and similarly, a higher proportion of students in this group had a screen sedentary time of greater than or equal to 2 h per day (*P* < 0.001) (see Table 3).

**TABLE 3:** Comparison of demographic characteristics of various dietary patterns in middle school students (*N* = 6,590).

Variables	Modern dietary pattern	Fast food dietary pattern	Balanced dietary pattern	*P*-value
	*N* = 2,280	*N* = 2,140	*N* = 2,170	
Age (years)	15 (14–16)	15 (14–16)	14 (14–15)	<0.001
Gender				0.140
Boys	1,146 (50.3%)	1,035 (48.4%)	1,027 (47.3%)	
Girls	1,134 (49.7%)	1,105 (51.6%)	1,143 (52.7%)	
Nationality				0.590
The Han	2,167 (95.0%)	2,028 (94.8%)	2,071 (95.4%)	
Others	113 (5.0%)	112 (5.2%)	99 (4.6%)	
School type				<0.001
Junior high school	2,006 (88.0%)	1,955 (91.4%)	2,013 (92.8%)	
Senior high school	274 (12.0%)	185 (8.6%)	157 (7.2%)	
Live in school or not				<0.001
Yes	1,061 (46.5%)	791 (37.0%)	826 (38.1%)	
No	1,219 (53.5%)	1,349 (63.0%)	1,344 (61.9%)	
Residence				<0.001
Towns	1,223 (53.6%)	1,278 (59.7%)	1,233 (56.8%)	
Country	1,057 (46.4%)	862 (40.3%)	937 (43.2%)	
Only children or not				0.700
Yes	511 (22.4%)	501 (23.4%)	490 (22.6%)	
No	1,769 (77.6%)	1,639 (76.6%)	1,680 (77.4%)	
Parenting mode				<0.001
Parenting by the parents’ generation	1,651 (72.4%)	1,685 (78.7%)	1,647 (75.9%)	
Grand parenting	582 (25.5%)	421 (19.7%)	485 (22.4%)	
Mixed parenting	47 (2.1%)	34 (1.6%)	38 (1.8%)	
Parenting education level				<0.001
Primary and below	716 (31.4%)	522 (24.4%)	525 (24.2%)	
Junior high school	1,288 (56.5%)	1,300 (60.7%)	1,342 (61.8%)	
High school/technical school	143 (6.3%)	194 (9.1%)	169 (7.8%)	
College/bachelor degree or above	133 (5.8%)	124 (5.8%)	134 (6.2%)	
Overweight/obesity or not				0.170
Yes	467 (20.5%)	396 (18.5%)	404 (18.6%)	
No	1,813 (79.5%)	1,744 (81.5%)	1,766 (81.4%)	
Body type self-assessment				<0.001
Lose weight	301 (13.2%)	239 (11.2%)	254 (11.7%)	
Normal	1,383 (60.7%)	1,446 (67.6%)	1,503 (69.3%)	
Overweight	313 (13.7%)	236 (11.0%)	230 (10.6%)	
Obesity	216 (9.5%)	161 (7.5%)	125 (5.8%)	
Other	67 (2.9%)	58 (2.7%)	58 (2.7%)	
Sleep sufficient or not				<0.001
Yes	2,209 (96.9%)	2,069 (96.7%)	2,057 (94.8%)	
No	71 (3.1%)	71 (3.3%)	113 (5.2%)	
Daily exercise time				0.030
<1 h/day	1,304 (57.2%)	1,249 (58.4%)	1,182 (54.5%)	
≥1 h/day	976 (42.8%)	891 (41.6%)	988 (45.5%)	
Daily screen sedentary time				
<2 h/day	1,062 (46.6%)	1,143 (53.4%)	1,270 (58.5%)	<0.001
≥2 h/day	1,218 (53.4%)	997 (46.6%)	900 (41.5%)	
External eating score	24 (19–27)	22 (19–26)	22 (18–27)	<0.001

### 3.4 Multiple linear regression analysis model of the association between external eating and dietary patterns in middle school students

The table analyzes the relationship between external eating scores and Modern dietary pattern, fast-food dietary pattern, and balanced dietary pattern, and establishes two models for analysis respectively. In model 1, external eating was negatively associated with modern dietary pattern (β = −0.007, 95% CI: −0.012, −0.003, *P* = 0.001), indicating that an increase in the score of external eating was associated with a decrease in the score of modern dietary pattern. This negative association remained significant after adjusting for confounding factors. Before adjustment for confounders, external eating was positively associated with the balanced dietary pattern (β = 0.005, 95% CI: 0.001, 0.009, *P* = 0.016), suggesting that higher scores of external eating were associated with higher scores of balanced dietary pattern. After adjusting for confounding factors, this positive association (β = 0.005, 95% CI: 0.001, 0.010, *P* = 0.016) remained significant (see Table 4).

**TABLE 4:** Multiple linear regression analysis model of the association between external eating and dietary patterns in middle school students.

Dietary patterns	Models	β	95% CI	*P*-value
Modern dietary pattern	Model 1	−0.007	−0.012, −0.003	0.001
	Model 2	−0.005	−0.009, −0.001	0.018
Fast food dietary pattern	Model 1	0.007	0.003, 0.011	0.001
	Model 2	0.006	0.002, 0.011	0.002
Balanced dietary pattern	Model 1	0.005	0.001, 0.009	0.016
	Model 2	0.005	0.001, 0.010	0.016

Model 1 was not adjusted. Model 2 adjusted age, school type, live in school or not, residence, parenting mode, parenting education level, body type self-assessment, sleep sufficient or not, and daily exercise time, daily screen sedentary time.

## 4 Discussion

Multiple studies (34–36) have shown that in China, there are three main dietary patterns among middle school students. The first is a diversified dietary pattern characterized by fruits, nuts, legumes, eggs, and dairy products. The second is a fast-food concentrated dietary pattern characterized by a higher intake of processed meats, seafood, sugary drinks, fried foods, and desserts. The third is a traditional dietary pattern characterized by a higher intake of vegetables and grains. In this study, we identified three types of dietary patterns, which are supported by published evidence. However, this study found that the modern dietary pattern among middle school students in Chongqing differs from previous studies. According to (37, 38) some studies, the modern dietary pattern, which is rich in pork, poultry, vegetables and seafood, and confectionery, has been found to be positively associated with generalized and centripetal obesity; but there are also healthy and sustainable types of modern dietary pattern; “The Nordic diet” (39) is a type of modern dietary pattern that emphasizes health and sustainability, encouraging the consumption of locally produced seasonal ingredients such as mushrooms, fish, and seafood, while also advocating for a reduction in the consumption of processed foods and sugary beverages. The characteristics of the Nordic diet include high intake of vegetables, fruits, whole grains, fish, low-fat dairy products, and legumes, and low intake of red meat, processed meats, sugary beverages, sweets, and refined grains. This finding is reminiscent of the characteristics of the modern dietary pattern discussed in this study. It highlights that adherence to a healthy and potentially sustainable Nordic diet is associated with favorable child development, thereby underscoring the significance of early diet on children’s long-term health (40). In addition, the characteristic of the “fast-food dietary pattern” is a large intake of fried dough foods, processed meat products, and sugary drinks. These foods are very popular among middle school students and are also more easily influenced by the smell and appearance of food. However, long-term adherence to this pattern has been linked to increased risks of obesity and metabolic disorders. The identified high prevalence of fast-food consumption among young adolescents in LMICs indicates (41) the urgent need to prioritize the implementation of healthy-diet promotion programs to improve adolescent health in these countries. Furthermore, the characteristic of the “balanced dietary pattern” is a large intake of grains, fresh vegetables, red meat, and purified water. In traditional Chinese diet, vegetables and grains play a core role in the dietary structure. These patterns reflect the complex interplay between traditional Chinese eating habits and modern dietary influences. Simultaneous research (42) indicates that individuals adhering to a balanced diet exhibit better brain health, cognitive function, and mental wellbeing, with superior memory and larger brain volume compared to those following sugar-controlled, vegetarian, or carnivorous diets. This study result also emphasizes (42) the importance of adopting a healthy lifestyle, including a balanced diet, from childhood through adolescence and into young adulthood, as these habits have a direct impact on health promotion and risk reduction. While the modern dietary pattern offers nutritional benefits, its limited adoption suggests cultural and accessibility barriers. The pervasive fast-food pattern, despite its health risks, continues to dominate due to its convenience and sensory appeal. The balanced pattern, though traditional, requires reinforcement through nutritional education to maintain its relevance among tech-savvy, modern middle school students. The results of this study show that the cumulative variance percentage is the highest for the balanced dietary pattern and the fast-food dietary pattern. It can be seen that the dietary pattern of middle school students is transitioning from the modern dietary pattern to the balanced dietary pattern and the fast-food dietary pattern.

In 2024, the disposable income of Chinese residents was 41,314 yuan, an increase of 5.1% compared with the previous year. The income growth has enabled low-income groups to prioritize meeting the demand for animal protein. While the high-income group turned to healthy alternatives (43). The dietary patterns of middle school students have undergone changes. On the one hand, the expansion of the food industry has enabled highly processed snacks such as potato chips and cola to permeate the lives of middle school students through campus convenience stores. The localization strategies of foreign fast food and the convenience of food delivery platforms have jointly driven the explosion of the fast food model. However, in the process of modernization, it has encountered the impact of globalization, triggering a dual conflict between cultural identity and health needs (44). With the increase of urbanization rate in Chongqing, the traditional heavy oil and salt dietary culture in Sichuan and Chongqing has a fundamental conflict with modern health demands. This transformation confirms the dynamic evolution law of dietary culture (45), achieving the transformation from the dietary framework of traditional culture to the dietary pattern of health identity. In the results of each item of external eating, there were no cases of “never” or “now and then” for the item “You can resist the temptation of delicious food.” This indicates that among middle school students, there is a strong sense of self-control and effective parental supervision. This phenomenon may be caused by a combination of various factors. First of all, it is important to note that parenting practices do not only affect the current eating habits of children but also influence how children choose their own food in the long term (46). In particular, a review (47) identified five parental characteristics—parental knowledge, attitudes, parenting style, time, and cost concern—as the underlying facilitators of or barriers to food parenting practices that influence students’ KAP on healthy eating. Scholars have pointed out that the family, as a place for health promotion, can significantly improve children’s healthy behaviors through setting rules and supervision (48). A study found (49) that self-compassion can buffer the negative impact of economic scarcity on healthy eating behaviors and self-control. Finally, the positive influence among classmates also plays an important role.

From the demographic characteristics of different dietary patterns among middle school students, it can be observed that urban students may have easier access to fast food, while rural students may rely more on traditional diets, with a higher proportion opting for balanced dietary pattern. A similar study (50) found that the dietary characteristics of rural males include higher consumption of grains and fats, while urbanization is associated with increased dairy consumption among females. One study (51) found that students raised by grandparents had significantly higher frequencies of fast food consumption compared to those raised by parents, primarily because grandparents tend to express affection by providing children with their preferred foods, such as fast food.

The results of the multiple linear regression analysis indicated that external eating was positively associated with balanced dietary pattern, which is inconsistent with previous studies. Some studies (52, 53) have shown that a balanced diet is easily affected by the outside world. Nowadays, people’s health awareness has been improved. The better the smell or appearance of healthy food, the stronger our desire to eat. Scholars have pointed out (54) that a healthy and sustainable dietary pattern needs to take into account both nutritional and environmental factors, and good appearance and smell are one of the important factors to attract consumers. It has been found (55) that product prices, the availability and convenience of food are important factors affecting students’ dietary behaviors. Future interventions aimed at improving external dietary behaviors should consider a multidimensional strategy. This includes enhancing policies to foster a healthier food environment at the national level, incorporating nutrition education into school curricula to guide students toward healthier food choices, and encouraging parents to model healthy eating behaviors to help establish positive dietary habits in middle school students.

The advantage of this paper lies in its large sample size, which includes middle schools from 17 districts and counties in Chongqing, and thus has good representativeness. Secondly, we collected information on the dietary behaviors and food intake frequency of middle school students, which allows us to have a comprehensive understanding of the current dietary habits and influencing factors of middle school students. Finally, the association between external eating and dietary patterns among middle school students was explored, providing valuable insights and evidence for future suggestions and interventions.

However, this article also has some limitations. First, this study collected questionnaire data through an online self-reported survey, which may be less effective than face-to-face interviews and could potentially lead to information bias. Second, due to the heavy study load of high school students, the number of high school students surveyed was significantly low. Moreover, students in schools in areas with stricter teaching management, such as the main urban area, are restricted in their use of mobile phones. Third, this study is a cross - sectional one, so it is not possible to make causal inferences between external eating and dietary patterns. Further longitudinal studies are needed in the future to provide evidence.

## 5 Conclusion

In conclusion, this study emphasizes the association between external eating and different dietary patterns among middle school students in Chongqing. Currently, there are three dietary patterns among middle school students: “modern dietary pattern,” “fast-food dietary pattern,” and “balanced dietary pattern.” The study also highlights the negative association between external eating and modern dietary pattern. To address the potential selection bias in future studies, we recommend employing a more rigorous sampling method. In future food and nutrition education, it is important to help and guide middle school students to correctly deal with external eating behaviors, promote balanced nutrition and health, and thus establish a reasonable dietary pattern.

## Data Availability

The raw data supporting the conclusions of this article will be made available by the authors, without undue reservation.
